# Livelihood disruption and psychological distress following the 2024 flash flood in Bangladesh

**DOI:** 10.1038/s41598-025-13418-0

**Published:** 2025-07-25

**Authors:** Md Mostafizur Rahman, Samantha Alam, Ifta Alam Shobuj, Md. Mehedi Hasan Santo, Md. Tanvir Hossain, Farzana Rahman, Edris Alam, Md Kamrul Islam

**Affiliations:** 1 Department of Disaster Management & Resilience, Faculty of Arts and Social Sciences, Bangladesh University of Professionals, Dhaka, 1216 Bangladesh; 2 Sociology Discipline, Social Science School, Khulna University, Khulna, 9208 Bangladesh; 3 Department of Computer Science and Engineering, Independent University, Bangladesh, Dhaka, 1205 Bangladesh; 4 Department of Geography and Environmental Studies, University of Chittagong, Chittagong, 4331 Bangladesh; 5 Faculty of Resilience, Rabdan Academy, Abu Dhabi, 22401 United Arab Emirates; 6 Department of Civil and Environmental Engineering College of Engineering, King Faisal University, AlAhsa, 31982 Saudi Arabia

**Keywords:** Flash flood, Livelihood disruption, Psychological distress, K10 scale, Bangladesh, Environmental social sciences, Natural hazards, Health care, Risk factors

## Abstract

The 2024 flash flood in Feni District, Bangladesh, caused substantial disruptions to livelihoods and posed serious mental health challenges for the affected population. This study aimed to assess the impact of the flood on both livelihood and psychological well-being using a cross-sectional design. Data were collected from 855 adult residents across three severely affected upazilas-Sonagazi, Chhagalnaiya, and Fulgazi. Livelihood impact was measured using a self-reported binary item, while psychological distress was assessed using the Kessler Psychological Distress Scale (K10). Descriptive and multiple regression analyses were conducted to identify key sociodemographic and flood-related predictors. Findings revealed that 99.3% of respondents experienced livelihood disruption, and over 85% reported moderate to severe psychological distress. Regression results indicated that education level, occupation, income, location, and chronic illness significantly influenced livelihood outcomes. Similarly, factors such as age, marital status, education, geographic location, family structure, and lack of early warning were significantly associated with psychological distress. Notably, limited access to safe drinking water and food scarcity during the flood exacerbated both livelihood and mental health impacts. These findings underscore the urgent need for integrated post-disaster interventions that address both economic recovery and mental health support. Enhancing early warning systems, improving resource accessibility, and strengthening community-based mental health services are critical to building resilience in flood-prone areas.

## Introduction

Floods are among the most common and destructive natural hazards globally, inflicting widespread damage to infrastructure, livelihoods, and human health^1,2^. In recent decades, climate change, deforestation, and rapid unplanned urban growth have intensified both the frequency and severity of floods^3^. Floods routinely cause deaths, injuries, displacement, and extensive economic losses. Annually, millions suffer, especially in developing countries where infrastructure is more vulnerable^4^.

Flash floods had profound consequences on the livelihoods of affected communities, particularly those dependent on agriculture, fisheries, and informal labour^5–7^. Floods could cause widespread damage to agricultural lands, leading to loss of crops, destruction of fishing gear, and disruption in local markets. As a result, many households might face income loss and food scarcity, exacerbating their vulnerability to long-term economic hardship. In particular, the rural poor, whose livelihoods are tied to agriculture and natural resource-based occupations, experienced significant reductions in income, often up to two-thirds of their usual earnings, which severely limited their ability to recover and prepare for future floods^6,7^.

Exposure to natural hazards is associated with a wide range of mental health issues, including posttraumatic stress disorder (PTSD), depression, anxiety, and prolonged emotional distress^8,9^. Recurrent floods undermine communities, altering livelihoods, eroding social structures, and disrupting daily life, all of which contribute to widespread psychosocial repercussions^4,10,11^. Beyond immediate trauma, well-documented consequences include increased rates of PTSD, depression, anxiety, and even suicide risk^4,8,12^. These effects may persist for months or years after the event.

In disaster management, it is crucial to adopt an intersectional framework to understand how various social conditions and experiences intersect to increase people’s vulnerability. Women, older adults, people with chronic illnesses, and those with limited social support are particularly at risk of experiencing heightened mental and economic distress^13,14^. Intersectionality highlights how overlapping identities, such as gender, class, and race, compound the effects of disasters on marginalized communities^14^. For example, women are more likely to report internalizing symptoms such as depression and anxiety, while men often manifest distress through aggression or substance use and are less likely to seek help^10,11^.

Bangladesh, situated in the delta of the Ganges, Brahmaputra, and Meghna rivers, is particularly susceptible to flooding due to its low-lying topography and dense population^10,15–17^. In low-resource settings like Bangladesh, the psychosocial toll of disasters is often compounded by poverty, inadequate access to mental health services, and stigma surrounding psychological disorders^18^. Women, older adults, people with chronic illnesses, and those with limited social support are particularly at risk^10,19^. Mental health issues were more prevalent among older, married, illiterate men, those living in temporary housing, and those working in agriculture or fishing^11^. Displacement, the loss of livelihood or loved ones, property damage, food insecurity, and inadequate warning or support systems are primary triggers for psychological distress^11,19,20^.

Furthermore, the disruption of livelihoods caused by floods, particularly among communities dependent on agriculture, fisheries, and informal labour, can intensify psychological distress. Studies in both developed and developing contexts have shown a strong link between socioeconomic insecurity and mental health outcomes following disasters^21,22^. However, in Bangladesh, comprehensive assessments that jointly examine the livelihood and mental health impacts of flash floods remain scarce.

In 2024, a sudden and severe flash flood struck the Feni District in southeastern Bangladesh. Unlike the more predictable seasonal monsoon floods, this event was abrupt and devastating, submerging entire villages, displacing thousands of people, and severely disrupting local economies^23,24^. The flood affected critical infrastructure and agricultural zones across Sonagazi, Chhagalnaiya, and Fulgazi Upazilas, exposing systemic vulnerabilities in disaster preparedness and response^25–27^. While immediate responses often focus on physical damage and economic loss, the mental health consequences of such disasters are less visible and frequently overlooked in disaster-affected communities in Bangladesh.

This study aims to fill this critical gap by evaluating the effects of the 2024 flash flood on both the livelihoods and psychological well-being of residents in the Feni District. Specifically, we assess the prevalence and severity of psychological distress using the Kessler Psychological Distress Scale (K10)^28^ and examine self-reported livelihood disruptions. Additionally, the study explores the role of sociodemographic and flood-related factors, such as education, income, geographic location, access to resources, and early warning systems, in shaping these outcomes.

By adopting a quantitative approach and surveying a large sample from three flood-affected upazilas, this research offers valuable insights for disaster response and recovery planning. Strengthening local resilience through disaster education, improved infrastructure, early warning systems, and accessible psychosocial support services is vital to reducing vulnerability and promoting sustainable recovery in flood-prone regions.

## Methods

### Research design

**Fig. 1:**
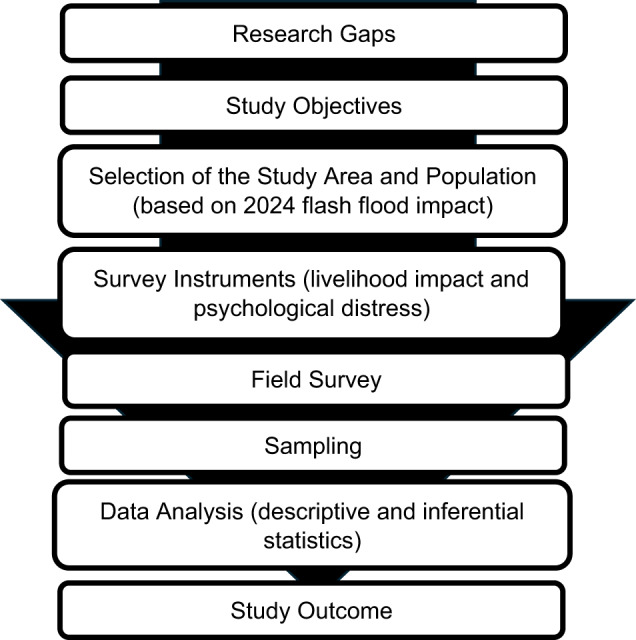
Flowchart of methodology.

**Fig. 2:**
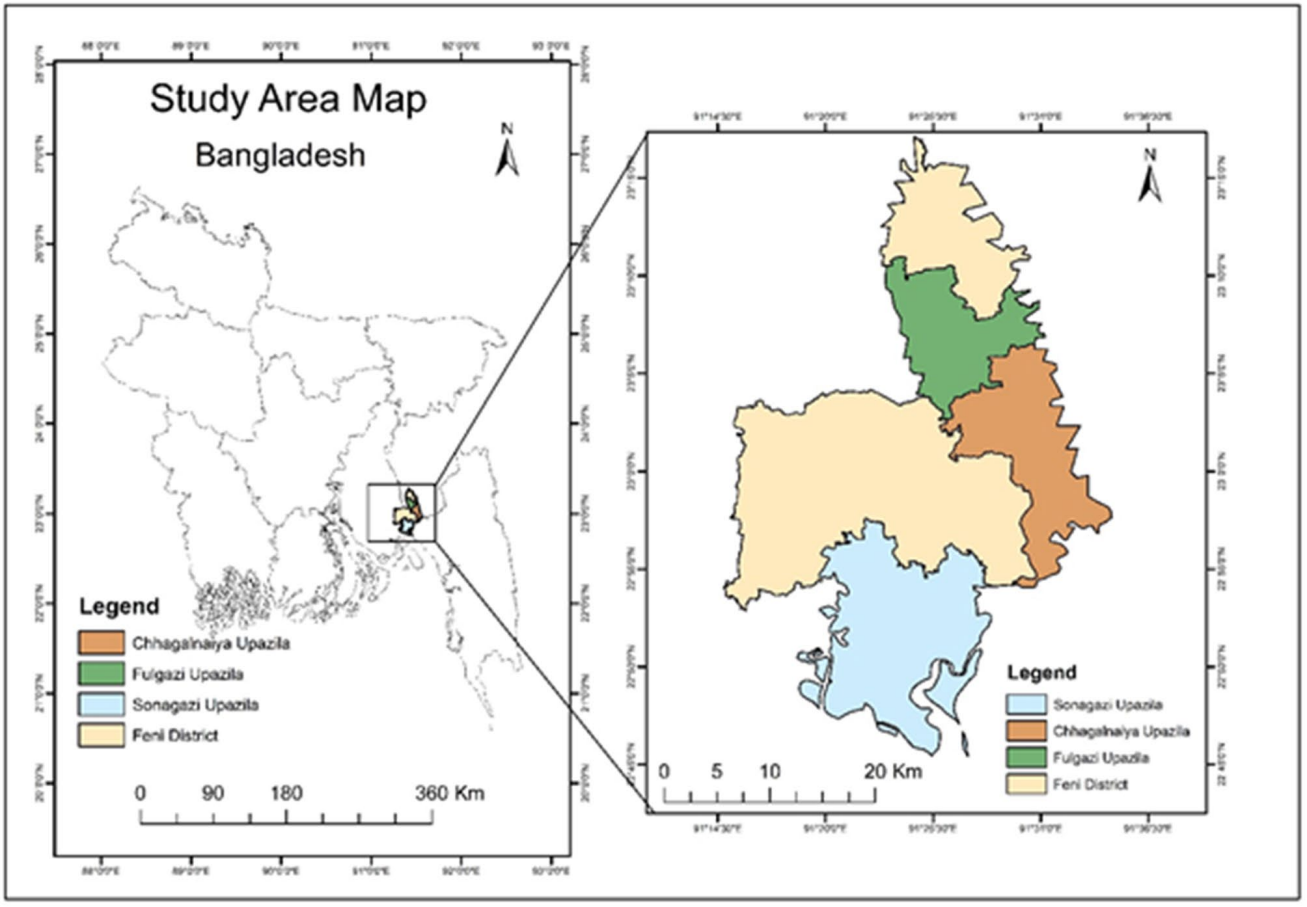
Study area.

This study employed a cross-sectional design to evaluate the impact of the 2024 flash flood on both livelihoods and mental health. Data were collected shortly after the flood from adult residents (aged 18 and above) across three affected Upazilas—Sonagazi, Chhagalnaiya, and Fulgazi—in the Feni District of Bangladesh (Figs. 1 and 2). Livelihood impact was measured using a self-reported binary question: “Did the 2024 flood impact your livelihood?” (Yes/No). Mental health status was evaluated using the Kessler Psychological Distress Scale (K10)^28^. Given the unprecedented nature of the flood, the study hypothesized that it could have led to significant socioeconomic disruptions and psychological distress among those affected.

### Study area

Bangladesh has a multi-tier administrative structure. Currently, the country is divided into 8 divisions, which are further subdivided into 64 districts. Each district is segmented into sub-districts called “Upazilas” (or sub-districts), and these are further divided into unions and villages. This hierarchical system enables localised governance and development planning.

Feni, formerly a subdivision of Noakhali District, was officially recognized as a separate district on March 1, 1984 ^29^. Geographically, it lies between latitudes 22˚44’ and 23˚17’ north and longitudes 91˚15’ and 91˚35’ east. The district is bordered to the north by Comilla District and India, to the east by India and Chattogram District, to the south by Chattogram and Noakhali Districts, and to the west by Noakhali District.

Fulgazi Upazila, located in the northern region of Feni, spans around 102.19 square kilometres^30^. It shares its eastern boundary with Tripura, India, and is known for its rich floodplain landscape, crisscrossed by rivers such as the Muhuri, Selonia, and Kahuya^31^. The upazila has a population of approximately 119,558 and a literacy rate close to 60% ^31^. Due to its low elevation and proximity to river systems, Fulgazi is highly vulnerable to flooding. Notably, the 1998 floods caused extensive damage to homes and agricultural land^32^and the 2024 floods affected more than 40 villages, displacing thousands^26,27^.

Chhagalnaiya Upazila, adjacent to Fulgazi, covers roughly 139.59 square kilometres and is home to about 187,156 residents^31^. Like Fulgazi, it shares a similar flood-prone terrain. In August 2024, intense rainfall and water runoff from India caused the local rivers to overflow, leading to severe flooding that submerged numerous villages and stranded many people^26,27^. The area’s reliance on agriculture further heightens the socioeconomic risks during such flood events.

Sonagazi Upazila, encompassing approximately 284.89 square kilometres and with a population of around 262,547, borders both Fulgazi and Chhagalnaiya^31^. It makes it part of a contiguous zone susceptible to flooding. In August 2024, flash floods affected nearly 350,000 people across the three upazilas, resulting in widespread disruption to daily life and infrastructure^25–27^. Flood events in this region frequently lead to submerged roads and limited access to vital services.

### Survey techniques

The survey was administered in Bengali, the native language of the respondents, and employed self-reported measures to evaluate the impact of the 2024 flood on both livelihood and mental health. 20 participants were involved in a pilot survey, which helped refine the final version of the questionnaire. Feedback from the pilot phase was used to improve the questionnaire’s layout and clarity. However, responses from the pilot were excluded from the primary dataset used in the final analysis. To assess the internal consistency of the K10 section, Cronbach’s alpha was calculated, with all subscales scoring above 0.75, indicating strong reliability. A Cronbach’s alpha value above 0.60 is typically considered acceptable for internal consistency^33,34^.

The finalized questionnaire comprised four core sections. The first section collected key sociodemographic details. The second section provided flood-related information, while the third focused on the impacts on livelihoods. The fourth section consisted of the Kessler 10 (K10) scale. Sociodemographic characteristics and flood-related information were treated as independent variables, posited to influence both livelihood disruption and levels of psychological distress measured through the K10. The survey was conducted face-to-face to ensure high response rates. After identifying the respondents, we distributed the sample evenly across the three upazilas (Sonagazi, Chhagalnaiya, and Fulgazi), taking into account the varying levels of flood exposure. Respondents from different socioeconomic backgrounds and occupations were included to ensure the findings reflect the diversity of the affected population. Data were collected through structured interviews using a questionnaire that included questions on sociodemographic characteristics, flood-related information, livelihood disruptions, and K10. The Bengali version of the K10 was used for this purpose^35^. Drawing on prior fieldwork in remote areas, the research team ensured that questions were simple and easily comprehensible. The following measurement tools were used:

#### Sociodemographic characteristics

The survey also included questions to collect sociodemographic details, such as gender, age, marital status, educational attainment, location, housing and family conditions, presence of vulnerable household members, and chronic health conditions.

#### Flood-related information

In addition to sociodemographic factors, the survey captured flood-related information to assess how these experiences might have influenced the respondents’ distress and livelihood disruption. These included: previous flood experience, safety of current residence, availability of resources for self-protection, socioeconomic support received during the flood, and access to early warning systems.

#### Livelihood disruption impact

Livelihood impact was measured using a self-reported binary question, “Did the 2024 flood impact your livelihood?” (Yes/No). This item was designed to provide a straightforward assessment of whether respondents experienced disruptions to their economic activities due to the flood. Given the intensity of the event, this measure aimed to determine whether individuals faced any challenges to their livelihoods due to the flood.

#### Kessler psychological distress scale

Psychological distress was assessed using the Kessler Psychological Distress Scale (K10)^28^. The K10 includes 10 questions designed to gauge the frequency of general psychological distress symptoms experienced during the previous four weeks. Participants were asked how often they felt: (1) unusually tired, (2) nervous, (3) extremely nervous and unable to calm down, (4) hopeless, (5) restless or uneasy, (6) so restless they couldn’t remain still, (7) sad or down, (8) so depressed that nothing could lift their mood, (9) overwhelmed by effort, and (10) worthless.

Responses were captured using a five-point Likert scale: none of the time (1), a little of the time (2), some of the time (3), most of the time (4), and all the time (5). The total K10 score was calculated by summing responses to all 10 items, yielding a possible range from 10 to 50. Based on this score, psychological distress levels were categorized into four tiers: likely to be well, likely mild disorder, likely moderate disorder, and likely severe disorder (Table 1). The K10 is a widely recognized tool for assessing emotional distress and has been used extensively in previous studies involving adult populations in Bangladesh^36–38^.

**Table 1:** Likelihood of having a mental disorder (psychological distress)^28^.

Likelihood	K10 Score range
Likely to be well	10–19
Likely to have a mild disorder	20–24
Likely to have a moderate disorder	25–29
Likely to have a severe disorder	30–50

### Data management

The field survey was conducted in February 2025. Participants’ flood exposure was assessed through screening items, such as self-reported experiences of physical injuries sustained during the event. The initial contact was made with a resident, who helped identify and reach households and individuals eligible for participation. This approach was necessary due to the constraints of accessing individuals in remote, flood-affected areas. The survey specifically targeted individuals who had directly experienced the 2024 flash flood in the selected study areas. To ensure the sample was representative, participants were selected from a diverse range of geographic locations, socioeconomic backgrounds, and flood exposure levels.

Given the unprecedented nature of the flash flood, a purposive sampling approach was adopted, specifically targeting those directly impacted by the event. Based on Morgan’s table, a minimum sample size of 384 was sufficient for statistical validity^39^. In addition, the sample size was also calculated following Yamane’s formula^40^:

$$\:n=\frac{N}{1+N\left({e}^{2}\right)}$$

where n = sample size, N = population, e = error tolerance.

The total population of Feni District is 1,437,371 ^31^. The required sample size was approximately 400 (with a 0.05 error tolerance). However, a larger sample of 855 respondents was used to provide a more granular understanding of the flood’s impact across different subgroups. This expansion enhances the robustness of the findings and provides a more precise estimate of the overall effects of the flood on both livelihoods and psychological well-being. The larger sample also facilitated more detailed subgroup analyses, such as those based on income, education, and geographic location.

Data processing and statistical analysis were carried out using Python (version 2.7; Beaverton, OR 97008, USA) and R (version 4.2.3)^41,42^. Descriptive statistics were computed to summarize key variables. Multiple logistic and linear regression models were employed to explore the relationships between sociodemographic and flood-related information and their impacts on livelihoods and psychological distress (K10 scores), respectively. Logistic regression was performed using the glm() function from base R, while linear regression was conducted using the lm() function from base R. Variables were initially screened through simple regression analyses, and multicollinearity was assessed using functions from the car package^43^ to ensure model robustness. Additionally, we have carefully selected the variables for use in simple regression analysis. For instance, gender was identified as a significant predictor of livelihood impact and was included in the final logistic regression model.

Livelihood disruption and K10 psychological distress scores were treated as dependent variables, while sociodemographic and flood-related information were considered independent variables. Accordingly, two separate multiple regression models, such as multiple logistic regression and multiple linear regression, were developed to analyze these relationships. In addition, K10 categories were computed to classify the severity of psychological distress, as detailed in Table 1.

## Results and discussion

### Sample profile

The majority of the respondents were male, accounting for 94.85% of the total sample (Table 2). It is reflective of the occupational characteristics in the region, where the most common livelihoods, particularly those affected by the flood, tend to be dominated by males. As the study specifically targeted these predominant livelihood activities, such as agro-fishery, business, and wage labor, which are traditionally male-dominated, the gender composition of the sample reflects the nature of these occupations. Most participants (57.43%) were aged between 36 and 55 years, while only 1.87% were aged 18–25. A large proportion (97.66%) were married. Regarding education, over half (54.39%) had completed up to non-SSC education, followed by 27.60% with SSC education, and a smaller percentage were illiterate (7.49%) or had more than SSC education (10.52%). In the context of this study, SSC refers to the Secondary School Certificate. It is a public examination taken by students in Bangladesh at the end of their 10th grade. The SSC is a significant milestone in Bangladesh’s education system, marking the completion of secondary education. Students who pass this examination are awarded the SSC certificate, which is required for further education, such as enrolling in higher secondary school (HSC) or vocational training. It is one of the key educational qualifications for young people in Bangladesh.

In terms of occupation, most respondents were involved in agro-fishery (44.44%), with other significant groups working in business (22.22%), wage labor (20.35%), and as employees (12.98%). The monthly income distribution showed that nearly half (49.24%) earned less than BDT 15,000, 46.67% earned between BDT 15,000 and BDT 29,999, and a small proportion (4.09%) earned more than BDT 30,000. Participants were from three upazilas: Fulgazi (34.27%), Sonagazi (34.04%), and Chhagalnaiya (31.7%). Most lived with their families (98.6%), and 85.26% had vulnerable family members. In this study, housing types were categorized into three groups, Kacha, Semi-pucca, and Pucca, based on construction quality and materials, which reflect residents’ socioeconomic status and vulnerability in flood-prone areas. Kacha houses, typically built with mud, bamboo, or thatch, are temporary and highly susceptible to flooding and other natural hazards, often indicating limited financial capacity. Pucca houses are constructed with durable materials like brick and concrete, offering greater resistance to environmental stress, and are more common among economically secure households. Semi-pucca houses combine elements of both, often featuring a brick or concrete base with less durable walls or roofs, providing moderate resilience. The prevalence of chronic disease and disability was low, with 91.93% reporting no chronic disease and 98.25% reporting no disability. In this study, chronic diseases refer to long-term health conditions that typically last for a year or more, require ongoing medical attention, or limit daily functioning. The study aimed to capture a broad range of such conditions that may increase participants’ vulnerability to flood-related impacts. Common examples include diabetes, hypertension, asthma, arthritis, heart disease, chronic respiratory conditions, and kidney disease. These illnesses can hinder individuals’ ability to respond to and recover from disasters by limiting mobility, increasing healthcare needs, and requiring continuous disease management. In this study, disability is defined as a broad range of physical, sensory, cognitive, or mental health impairments that may limit an individual’s ability to carry out daily activities or participate fully in social and economic life. The focus was on disabilities that could affect a person’s capacity to respond to and cope with flood-related challenges. Examples include physical impairments (e.g., mobility limitations, paralysis) and sensory disabilities (e.g., visual or hearing impairments). These forms of disability, recognized by both health and social systems, can significantly shape individual resilience and vulnerability in disaster contexts.

**Table 2:** Sociodemographic information.

Features	Frequency (Percentage %)
1. Gender	
*Male*	*811 (94.85)*
*Female*	*44 (5.15)*
2. Age group (year)	
*18–25*	*16 (1.87)*
26–35	147 (17.19)
36–45	244 (28.54)
46–55	247 (28.89)
> 55	201 (23.51)
3. Marital status	
*Married*	*835 (97.66)*
*Unmarried*	*20 (2.34)*
4. Education	
*Illiterate*	*64 (7.49)*
*Non-SSC*	*465 (54.39)*
*SSC*	*236 (27.60)*
*More than SSC*	*90 (10.52)*
5. Occupation	
*Agro-fishery*	*380 (44.44)*
*Business*	*190 (22.22)*
*Wage Labor*	*174 (20.35)*
*Employee*	*111 (12.98)*
*6. Monthly Income (BDT)*	
*Less than 15*,*000 (less than 123 USD)*	*421 (49.24)*
*15*,*000–29*,*999 (around 123 USD – 247 USD)*	*399 (46.67)*
*30*,*000–49*,*999 (around 247 USD – 412 USD)*	*35 (4.09)*
*7. Upazila*	
*Fulgazi*	*293 (34.27)*
*Sonagazi*	*291 (34.04)*
*Chhagalnaiya*	*271 (31.7)*
8. Living with Family	
*Yes*	*843 (98.6)*
*No*	*12 (1.4)*
9. Housing type	
*Kacha*	*481 (56.26)*
*Pucca*	*135 (15.79)*
*Semi-pucca*	*239 (27.95)*
10. Vulnerable family member (child, pregnant woman, older person, etc.)	
*Yes*	*729 (85.26)*
*No*	*126 (14.74)*
11. Chronic disease	
*Maybe*	*42 (4.91)*
*No*	*786 (91.93)*
*Yes*	*27 (3.16)*
12. Disability	
*No*	*840 (98.25)*
*Yes*	*15 (1.75)*

### Flood-related information

The findings reveal a comprehensive picture of the respondents’ exposure, vulnerability, and experiences during the 2024 flash flood in the Feni district (Table 3). A significant majority (77.66%) reported no prior experience with flooding, underscoring the unprecedented nature of this disaster for most individuals. It aligns with the Rapid Needs Assessment report, which noted that 95% of the population in Fulgazi and Feni Sadar Upazilas were severely affected, with 90% of shelters submerged under water depths of 3–7 feet^44^. Despite 84.68% of respondents perceiving their residences as moderately safe, only 0.12% considered them completely safe, reflecting systemic infrastructural vulnerabilities. Alarmingly, 89% lacked resources to protect themselves, paralleling findings in Noakhali, where similar resource shortages were reported^45^.

The economic impact was severe, with nearly all respondents (99.3%) experiencing income loss due to the flood. It is consistent with reports highlighting damaged markets and disrupted livelihoods across affected districts^44,45^. However, only 42.22% received socioeconomic support during the event, highlighting gaps in relief distribution mechanisms. The absence of early warning systems (98.25%) and dissemination mechanisms (93.22%) further exacerbated vulnerabilities, echoing findings from other studies emphasizing the critical role of timely warnings in disaster preparedness^46^.

Flood duration varied, but most respondents (82.57%) faced inundation for 7–10 days, with nearly all houses affected (99.88%). Access to necessities was severely disrupted—92.28% lacked safe drinking water, and 85.5% faced food scarcity during the flood. These findings align with assessments from Feni town, where water sources were destroyed and food stocks depleted^47^. Despite these challenges, 80% evacuated to shelters, although overcrowding likely compounded health risks.

Health impacts were multifaceted: while personal injuries were reported by only 1.87%, disease contraction affected 8.65%, and illness within families impacted 36.26%. These figures are consistent with reports of emerging waterborne diseases in flood-affected areas due to damaged sanitation facilities^44^. The findings underscore the intersection between physical health risks and systemic failures in sanitation and hygiene infrastructure.

**Table 3:** Flood-related information.

Features	Frequency (Percentage%)
1. Previous flood experience before the recent flood	
*No*	664 (77.66)
*Yes*	191 (22.34)
2. Current place’s safety rating against flood	
*Moderately Safe*	724 (84.68)
*Unsafe*	130 (15.2)
*Safe*	1 (0.12)
3. I do not have the resources to protect myself from my exposure to floods	
*Agree*	767 (89.00)
*Neutral*	86 (10.06)
*Disagree*	2 (0.23)
4. Has your income been affected due to the 2024 flood?	
*Yes*	849 (99.3)
*No*	6 (0.7)
5. Did you get any kind of socioeconomic support in the last few days during the flood?	
*No*	494 (57.78)
*Yes*	361 (42.22)
6. Did you receive an early warning regarding the flood?	
*No*	840 (98.25)
*Yes*	15 (1.75)
7. How would you rate the early warning mechanism for floods in your locality?	
*Insufficient*	58 (6.78)
*No Early Warning Dissemination Mechanism at All*	797 (93.22)
8. What was the duration of the recent flood in your locality?	
*2–3 Days*	2 (0.23)
*4–6 Days*	118 (13.8)
*7–10 Days*	706 (82.57)
*11 Days or More*	29 (3.39)
9. Had your house been inundated during the recent flood?	
*No*	1 (0.12)
*Yes*	854 (99.88)
10. Did you have access to safe drinking water during the flood?	
*No*	789 (92.28)
*Yes*	66 (7.72)
11. Did you face any type of food scarcity to provide food for your family during the recent flood?	
*No*	124 (14.5)
*Yes*	731 (85.5)
12. Did you evacuate to the shelter during the flood?	
*No*	171 (20.0)
*Yes*	684 (80.0)
13. Have you been injured due to the flood?	
*Yes*	16 (1.87)
*No*	839 (98.13)
14. Have you got any diseases due to the flood?	
*Yes*	74 (8.65)
*No*	781 (91.35)
15. Have any family members injured during the recent flood?	
*Yes*	65 (7.6)
*No*	790 (92.4)
16. Have any family members experienced the disease during the recent flood?	
*Yes*	310 (36.26)
*No*	545 (63.74)

### Psychological distress

The assessment of psychological distress among the respondents revealed a concerning level of mental health burden following the 2024 flash flood in the Feni district. As shown in Table 4, only a very small proportion of participants (0.58%) were likely to be well, while the vast majority exhibited varying degrees of psychological distress. Over 85% of participants exhibited moderate to severe symptoms of distress, with 41.06% likely suffering from severe disorders. It aligns with broader observations from disaster-affected regions, where rapid-onset floods often lead to significant mental health challenges due to factors like limited forewarning, disruption of livelihoods, and loss of social support^10,48–52^. Approximately 13.69% were likely to have a mild mental disorder, 44.67% were likely to have a moderate disorder, and a striking 41.06% were likely to have a severe disorder. The distress levels in Feni mirror global patterns observed in post-disaster scenarios. The findings emphasize an urgent need for targeted mental health support and community-based interventions. Lessons from other regions suggest that protective measures like social support networks and accessible healthcare can mitigate long-term impacts.

**Table 4:** Likelihood of having a mental disorder (psychological distress).

Likelihood	*n* (%)
Likely to be well	5 (0.58%)
Likely to have a mild disorder	117 (13.69%)
Likely to have a moderate disorder	382 (44.67%)
Likely to have a severe disorder	351 (41.06%)

### Associated factors

Table 5 (see supplementary file) and Table 6 present the results of the simple and multiple regression analyses, respectively, identifying several key factors significantly associated with livelihood impacts (Model I) and psychological impacts (Model II) resulting from flash floods in the Feni district. Logistic regression was employed for Model I, while linear regression was used for Model II. Respondents with non-SSC level education had significantly higher odds of livelihood disruption compared to illiterate individuals (aOR = 3.47, 95% CI: 1.47–7.91). It suggests that partial education may not provide sufficient skills or resources to mitigate flood impacts. Individuals might lack the vocational or technical skills needed for flood mitigation and recovery, which is important to reduce the impact of the flood^53^. It also indicates that the education level influences flood preparedness and resilience. For instance, a study in the Tanguar Haor region found that education significantly affects flood preparedness, with individuals having higher education levels being better equipped to handle flood situations^54^.

**Table 5:** Factors associated with the flood impact on livelihood and mental health (Simple regression analysis).

Features	Model I^a^			Model II^b^		
	Livelihood affected			Psychological Distress		
	(OR^#^ (95% CI))	Standard Error	z value	(β^##^ (95% CI))	Standard Error	t value
**Gender**						
Male	2.49 (1.31, 4.63)**	0.31	2.87	0.20 (−0.86, 1.27)	0.54	0.37
Female	Reference			Reference		
**Age group (year)**						
18–25	Reference			Reference		
26–35	0.52 (0.11, 1.71)	0.66	−0.97	2.77 (0.97, 4.57)**	0.91	3.03
36–45	0.69 (0.15, 2.23)	0.65	−0.55	3.54 (1.78, 5.30)***	0.89	3.94
46–55	0.75 (0.16, 2.43)	0.65	−0.43	3.26 (1.50, 5.02)***	0.89	3.63
> 55	1.48 (0.32, 4.98)	0.67	0.59	2.88 (1.11, 4.65)**	0.90	3.19
**Marital status**						
Married	Reference			Reference		
Unmarried	0.67 (0.26, 1.93)	0.49	−0.78	−3.14 (−4.69, - −1.60)***	0.78	−3.99
**Education**						
Illiterate	Reference			Reference		
Non-Secondary School Certificate	1.85 (0.93, 3.47)**	0.33	1.86	−1.08 (−1.99, −0.17)**	0.46	−2.34
SSC (Secondary School Certificate)	0.75 (0.37, 1.42)	0.33	−0.84	−1.40 (−2.27, −0.50)**	0.49	−2.86
More than SSC	0.17 (0.08, 0.36)***	0.37	−4.64	−2.11 (−2.37, −0.44)***	0.56	−3.71
**Occupation**						
Agro-fishery	Reference			Reference		
Business	0.40 (0.26, 0.61)***	0.21	−4.23	−0.19 (−0.80, 0.41)	0.31	−0.63
Wage Labor	2.14 (1.17, 4.19)*	0.32	2.35	−0.34 (−0.97, 0.28)	0.32	−1.06
Employee	0.10 (0.06, 0.17)***	0.24	−9.12	−0.71 (−1.46, 0.02)	0.37	−1.90
**Monthly Income (BDT)**						
Less than 15,000 (less than 123 USD)	Reference			Reference		
15,000–29,999 (around 123 USD – 247 USD)	1.38 (0.98, 1.96)	0.17	1.86	0.7 (0.25, 1.20)**	0.24	2.99
30,000–49,999 (around 247 USD – 412 USD)	0.03 (0.01, 0.09)***	0.54	−6.02	−0.66 (−1.86, 0.54)	0.61	−1.07
**Upazila**						
Chhagalnaiya	Reference			Reference		
Sonagazi	17.77 (9.47, 37.15)***	0.34	8.33	1.46 (0.91, 2.00)***	0.27	5.28
Fulgazi	1.71 (1.20, 2.44)**	0.18	2.97	− 1.60 (−2.15, −1.06)***	0.27	−5.83
**Living with Family**						
Yes	4.91 (1.55, 16.76)**	0.59	2.69	4.23 (2.24, 6.21)***	1.01	4.18
No	Reference			Reference		
**Housing Type**						
Kacha	Reference			Reference		
Pucca	0.23 (0.15, 0.35)***	0.21	−6.76	−0.86 (−1.53, −0.19)*	0.34	−2.54
Semi-pucca	0.53 (0.36, 0.77)**	0.19	−3.25	−0.07 (−0.61, 0.46)	0.27	−0.26
**Vulnerable Family Member**						
Yes	1.89 (1.24, 2.85)**	0.20	3.05	−0.64 (−1.30, 0.02)	0.33	−1.89
No	Reference			Reference		
**Chronic Disease**						
No	10.94 (5.54, 23.21)***	0.36	6.61	−3.90 (−4.96, −2.84)***	0.54	−7.23
Maybe	Reference			Reference		
Yes	35.22 (8.64, 242.99)***	0.81	4.37	−4.03 (−5.68, −2.38)**	0.84	−4.79
**Previous flood experience before the recent flood**						
No	Reference			Reference		
Yes	0.83 (0.58, 1.22)	0.19	−0.91	−1.72 (−2.27, −1.16)***	0.28	−6.10
**Did you get any kind of socioeconomic support in the last few days during the flood?**						
No	Reference			Reference		
Yes	1.75 (1.25, 2.47)**	0.17	3.26	−0.93 (−1.40, −0.45)***	0.24	−3.86
**Did you receive an early warning regarding the flood?**						
Yes	0.04 (0.00, 0.15)***	0.76	−4.14	5.81 (4.06, 7.56)***	0.89	6.51
No	Reference			Reference		
**How would you rate the early warning mechanism for floods in your locality?**						
Insufficient	Reference			Reference		
No Early Warning Dissemination Mechanism at All	0.98 (0.49, 1.81)	0.32	−0.05	2.58 (1.65, 3.50)***	0.46	5.49
**Did you have access to safe drinking water during the flood?**						
Yes	0.23 (0.14, 0.40)***	0.26	−5.46	−1.72 (−2.59, −0.84)***	0.44	−3.86
No	Reference			Reference		
**Did you face any type of food scarcity to provide food for your family during the recent flood?**						
No	Reference			Reference		
Yes	4.35 (2.92, 6.49)***	0.20	7.22	−0.21 (−0.8, 0.45)	0.34	−0.62
**Did you evacuate to the shelter during the flood?**						
No	Reference			Reference		
Yes	3.35 (2.33, 4.80)***	0.18	6.56	0.27 (−0.30, 0.86)	0.30	0.93
**Have you been injured due to the flood?**						
Yes	0.22 (0.07, 0.59)**	0.51	−2.96	−4.69 (−6.40, −2.98)***	0.87	−5.39
No	Reference			Reference		
**Have you got any diseases due to the flood?**						
Yes	1.07 (0.61, 1.96)	0.29	0.23	−0.93 (−1.77, −0.09)*	0.42	−2.19
No	Reference			Reference		
**Family Member Injured During 2024 Flood**						
Yes	1.07 (0.59, 2.05)	0.31	0.23	−4.23 (−5.07, −3.39)***	0.42	−9.87
No	Reference			Reference		
**Family Member Experienced Disease During 2024 Flood**						
Yes	1.80 (1.27, 2.59)**	0.18	3.25	0.42 (−0.06, 0.91)	0.24	1.70
No	Reference			Reference		

*p < 0.05; **p < 0.01; ***p < 0.001; OR^#^ = Odds Ratio; β^##^ = Beta (Coefficient). The beta coefficient indicates how much the outcome variable varies for every one-unit variation in the predictor variable (Swinscow & Campbell, 2002). CI = Confidence Interval. Model I^a^ = Simple logistic regression analysis, Model II^b^ = Simple linear regression analysis.

In terms of occupation, those involved in business (aOR = 0.32, 95% CI: 0.18–0.56) and employed individuals (aOR = 0.12, 95% CI: 0.05–0.25) were significantly less likely to experience livelihood impacts compared to those in agro-fishery. It suggests that diversification away from agriculture can enhance resilience.​ It was reported that agricultural wages declined by 5% in flood-prone areas and 14% in severely exposed areas during the 1998 extreme floods in Bangladesh^55^. Long-term impacts were more severe, with wage losses persisting for over five years. Another study conducted after the flash flood in Cox’s Bazar, Bangladesh, found that poverty and precarious livelihoods exacerbated the impacts, forcing affected households to take loans, sell assets, and migrate^56^. Another study conducted in the southeast of Bangladesh found that the farmers faced the highest relative flood damage costs (35% of income), followed by fishermen (32%)^57^. A study in West Bengal highlights challenges such as limited access to resources and social constraints affecting agricultural labourers’ ability to diversify into higher-value occupations^58^. A study considered the Sylhet Haor Basin of Bangladesh, where flash floods severely affected agricultural livelihoods, prompting many to shift to non-agricultural occupations^59^.​.

Households with a monthly income of BDT 30,000–49,999 had significantly lower odds of livelihood impact (aOR = 0.14, 95% CI: 0.03–0.43) than those earning BDT 15,000–29,999. It underscores the protective effect of higher income against flood-related disruptions.​ It is consistent with research indicating that higher income levels enable better preparedness and recovery from floods. For example, in the Jamuna floodplain of Bangladesh, households with higher incomes were better able to cope with and adapt to flooding events^60^. Income inequality exacerbates vulnerability; policies promoting equality could reduce flood damage costs.

Residence in Sonagazi Upazila was associated with a notably higher likelihood of livelihood disruption (aOR = 11.66, 95% CI: 5.51–27.15) compared to Chhagalnaiya. It highlights the role of geographic location in flood vulnerability.​ Chronic illness was a strong predictor, with individuals reporting chronic disease showing a dramatically increased risk (aOR = 87.84, 95% CI: 14.58–788.21) relative to the ‘maybe’ category. It emphasizes the compounded vulnerability faced by individuals with health issues during floods.​ While specific studies on chronic illness and flood impact are limited, research indicates that health challenges exacerbate the difficulties in coping with flood events, especially among the rural poor^7^.​ Additionally, having access to safe drinking water during the flood was associated with reduced odds of livelihood impact (aOR = 0.47, 95% CI: 0.23–0.97), while experiencing food scarcity during the flood significantly increased the likelihood of impact (aOR = 2.64, 95% CI: 1.52–4.55).

In Model II, several factors were significantly associated with psychological distress among flood-affected individuals in the Feni district (Table 6). Individuals aged 26–55 years experienced significantly higher levels of distress compared to the 18–25 age group, with β coefficients ranging from 1.77 to 1.88. A systematic review of post-natural hazard mental health in Bangladesh identified age as a significant demographic factor influencing mental health outcomes, noting that middle-aged individuals often face increased responsibilities and stressors during disasters^9^. Research shows that psychological distress tends to decline with age, particularly from early adulthood to older age. However, middle-aged adults often report higher distress levels due to exposure to specific stressors, such as work crises or negative social relationships^61^. Middle adulthood is associated with unique psychosocial challenges, such as economic precarity, caregiving responsibilities, and chronic stress exposure, which may explain elevated distress in this group^62^.

Unmarried respondents reported significantly lower levels of psychological distress than their married counterparts (β = −1.90, 95% CI: −3.28 to −0.52). Research has shown that marital status is significantly correlated with depression, with married individuals often experiencing higher levels of stress and anxiety during disasters due to concerns about family safety and well-being^9^.

Education was inversely associated with psychological distress; those with non-SSC (β = −1.16), SSC (β = −1.38), and more than SSC education (β = −1.90) reported significantly less distress than illiterate individuals. Studies have demonstrated that individuals with lower levels of education exhibit higher levels of mental health symptoms, including anxiety and depression, during and after natural hazards^9^. Lower education is often associated with reduced health literacy, limited access to preventive care, and higher baseline stress—all of which may amplify disaster-related anxiety and depression.

Residence in Sonagazi Upazila was associated with higher psychological distress (β = 1.29, 95% CI: 0.74 to 1.83), and those living with family also reported higher distress levels (β = 3.39, 95% CI: 1.64 to 5.14). Studies highlight caregiver burden and amplified stress when managing family safety during disasters^63^.

Chronic illness showed a significant inverse association; individuals without chronic disease (β = −2.44) and those with chronic disease (β = −2.30) both reported lower levels of distress compared to those uncertain about their chronic disease status. Generally, health-related factors, including physical injury and disability during natural hazards, are associated with increased mental health problems. The lower distress levels among individuals with chronic illness in our study may warrant further investigation to understand the underlying causes.

Receiving socioeconomic support was also associated with reduced psychological distress (β = −0.65, 95% CI: −1.09 to −0.20). The association between receiving socioeconomic support and reduced psychological distress aligns with findings that social support is a critical factor in mitigating mental health issues during disasters^64^. Access to financial assistance and community support networks can alleviate stress and promote resilience^64,65^.​.

The early warning had a strong positive association with psychological distress (β = 3.93, 95% CI: 2.19 to 5.67), and the perception of having no early warning dissemination mechanism significantly increased distress levels (β = 2.93, 95% CI: 2.01 to 3.86) compared to those who rated the system as insufficient. Access to safe drinking water during the flood was protective (β = −1.26, 95% CI: −2.03 to −0.49). The protective effect of access to safe drinking water during floods on psychological distress is supported by studies highlighting the importance of essential resources in mitigating mental health issues during disasters^9^.

Interestingly, individuals who were physically injured during the flood experienced significantly less psychological distress (β = −2.94, 95% CI: −4.43 to −1.46), while those who became ill due to the flood had higher distress levels (β = 1.46, 95% CI: 0.64 to 2.27). Finally, having a family member injured during the 2024 flood was strongly associated with reduced psychological distress (β = −3.61, 95% CI: −4.37 to −2.85). The association of having a family member injured during the 2024 flood with reduced psychological distress is unexpected. Typically, the loss or injury of family members during disasters is linked to higher levels of depression and anxiety. This discrepancy suggests the need for further research to explore coping mechanisms and cultural factors influencing these outcomes.

**Table 6:** Associated factors with the flood impact on livelihood and mental health.

Features	Model I^a^			Model II^b^		
	Livelihood affected			Psychological Distress		
	(aOR^#^ (95% CI))	Standard Error	z value	(β^##^ (95% CI))	Standard Error	t value
**Gender**						
Male	1.18 (0.47, 2.89)	0.46	0.36			
Female	Reference					
**Age group (year)**						
18–25				Reference		
26–35				1.88 (0.36, 3.41)*	0.78	2.42
36–45				1.79 (0.25, 3.33)*	0.79	2.28
46–55				1.77 (0.22, 3.32)*	0.79	2.25
> 55				1.22 (−0.35, 2.8)	0.8	1.52
**Marital status**						
Married				Reference		
Unmarried				−1.90 (−3.28, −0.52)**	0.70	−2.71
**Education**						
Illiterate	Reference			Reference		
Non-SSC	3.47 (1.47, 7.91)**	0.43	2.92	−1.16 (−1.93, −0.38)**	0.39	−2.93
SSC (Secondary School Certificate)	2.42 (0.95, 6.06)	0.47	1.88	−1.38 (−2.27, −0.50)**	0.45	−3.07
More than SSC	2.45 (0.78, 7.78)	0.59	1.53	−1.90 (−3.00, −0.80)***	0.56	−3.4
**Occupation**						
Agro-fishery	Reference					
Business	0.32 (0.18, 0.56)***	0.3	−3.91			
Wage Labor	1.41 (0.70, 2.99)	0.37	0.94			
Employee	0.12 (0.05, 0.25)***	0.39	−5.5			
**Monthly Income (BDT)**						
Less than 15,000 (less than 123 USD)	Reference			Reference		
15,000–29,999 (around 123 USD – 247 USD)	0.61 (0.35, 1.06)	0.28	−1.72	0.28 (−0.20, 0.76)	0.25	1.13
30,000–49,999 (around 247 USD – 412 USD)	0.14 (0.03, 0.43)**	0.64	−3.12	−0.28 (−1.38, 0.83)	0.56	−0.5
**Upazila**						
Chhagalnaiya	Reference			Reference		
Sonagazi	11.66 (5.51, 27.15)***	0.4	6.08	1.29 (0.74, 1.83)***	0.28	4.65
Fulgazi	1.07 (0.66, 1.72)	0.24	0.27	−0.58 (−1.19, 0.02)	0.31	−1.89
**Living with Family**						
Yes	0.53 (0.11, 2.42)	0.77	−0.82	3.39 (1.64, 5.14)***	0.89	3.80
No	Reference			Reference		
**Housing Type**						
Kacha	Reference			Reference		
Pucca	1.26 (0.57, 2.80)	0.41	0.58	0.17 (−0.48, 0.81)	0.33	0.51
Semi-pucca	1.11 (0.64, 1.94)	0.28	0.37	0.27 (−0.22, 0.77)	0.25	1.09
**Vulnerable Family Member**						
Yes	0.90 (0.48, 1.65)	0.32	−0.35			
No	Reference					
**Chronic Disease**						
No	17.53 (5.62, 58.99)***	0.6	4.79	−2.44 (−3.53, −1.35)***	0.56	−4.39
Maybe	Reference			Reference		
Yes	87.84 (14.58, 788.21)***	0.99	4.51	−2.30 (−3.82, −0.77)**	0.78	−2.95
**Previous flood experience before the recent flood**						
No				Reference		
Yes				−0.32 (−0.93, 0.29)	0.31	−1.04
**Did you get any kind of socioeconomic support in the last few days during the flood?**						
No	Reference			Reference		
Yes	1.43 (0.14, 11.04)	0.23	−0.01	−0.65 (−1.09, −0.20)**	0.23	−2.85
**Did you receive an early warning regarding the flood?**						
Yes	1.43 (0.14, 11.04)	1.08	0.32	3.93 (2.19, 5.67)***	0.89	4.44
No	Reference			Reference		
**How would you rate the early warning mechanism for floods in your locality?**						
Insufficient				Reference		
No Early Warning Dissemination Mechanism at All				2.93 (2.01, 3.86)***	0.47	6.25
**Did you have access to safe drinking water during the flood?**						
Yes	0.47 (0.23, 0.97)*	0.36	−2.06	−1.26 (−2.03, −0.49)**	0.39	−3.2
No	Reference			Reference		
**Did you face any type of food scarcity to provide food for your family during the recent flood?**						
No	Reference					
Yes	2.64 (1.52, 4.55)***	0.27	3.48			
**Did you evacuate to the shelter during the flood?**						
No	Reference					
Yes	1.78 (0.89, 3.53)	0.35	1.65			
**Have you been injured due to the flood?**						
Yes	0.49 (0.10, 2.45)	0.81	−0.88	−2.94 (−4.43, −1.46)***	0.76	−3.89
No	Reference			Reference		
**Have you got any diseases due to the flood?**						
Yes				1.46 (0.64, 2.27)***	0.42	3.5
No				Reference		
**Family Member Injured During 2024 Flood**						
Yes				−3.61 (−4.37, −2.85)***	0.39	−9.36
No				Reference		
**Family Member Experienced Disease During 2024 Flood**						
Yes	0.88 (0.54, 1.43)	0.24	−0.53			
No	Reference					

*p < 0.05; **p < 0.01; ***p < 0.001; *aOR*^*#*^ = Adjusted Odds Ratio; *β*^*##*^ = Beta (Coefficient). The beta coefficient indicates how much the outcome variable varies for every one-unit variation in the predictor variable^66^. CI = Confidence Interval. Model I^*a*^ = Multiple logistic regression analysis, Model II^*a*^ = Multiple linear regression analysis.

### Limitations and strengths

Despite offering essential insights, this study has several limitations that warrant consideration.

First, the study used a single binary question to measure livelihood disruption. While this approach provides a broad overview, it may not fully capture the complex and multifaceted nature of livelihood impacts. Different livelihoods (e.g., agriculture, fishing, wage labor) may have been affected in various ways, and a more detailed set of questions could provide a richer understanding of the specific challenges faced by respondents. Future research could benefit from using more granular and multi-dimensional questions to explore the particular types of livelihood disruptions. Second, the cross-sectional nature of the study limits the ability to infer causal relationships between flood exposure and the observed outcomes of livelihood and mental health. Longitudinal follow-up would be more effective in capturing the progression of psychological distress and recovery of livelihood over time. Third, the purposive sampling strategy, although practical in post-disaster settings, may have introduced selection bias that potentially overrepresents those who are more accessible or more severely affected, thereby limiting the generalizability of the results to the broader population. Fourth, this study relied on self-reported data, particularly concerning psychological distress, which could be subject to recall bias. However, to mitigate this, we collected data shortly after the flood, thereby reducing the likelihood that participants would inaccurately recall their experiences. By gathering data promptly, we aimed to capture the immediate psychological and livelihood impacts, which enhances the reliability of the findings and provides a more accurate representation of the event’s immediate effects. Additionally, crucial confounding variables, such as pre-existing mental health conditions, coping strategies, and access to mental health or relief services before the disaster, were not fully captured. Finally, the geographic focus on the Feni District restricts the study’s applicability to other flood-prone regions in Bangladesh or areas with different environmental, infrastructural, and cultural contexts.

Nonetheless, the study possesses several key strengths. It addresses a significant research gap by jointly examining the effects of flash flooding on both livelihoods and mental health in a highly vulnerable region. The use of a widely validated tool, K10, enhances reliability and facilitates meaningful comparisons with other research. The large sample size improves the statistical robustness of the findings and supports subgroup analyses across diverse sociodemographic characteristics. Moreover, the study identifies multiple determinants- such as education, occupation, income, location, and access to basic needs—that can guide targeted interventions and inform disaster risk reduction strategies. By shedding light on the dual burden of economic and psychological distress following a sudden-onset disaster, this research provides a valuable foundation for developing holistic response frameworks and strengthening community resilience in Bangladesh and similar settings.

### Recommendations

Based on the findings of this study, several actionable recommendations can be proposed to enhance disaster preparedness, livelihood resilience, and mental health support for flood-affected communities in Bangladesh:

## 1. Integrate mental health into disaster response planning

Mental health services should be an essential component of disaster management strategies. Local health authorities and NGOs should incorporate psychological first aid, trauma counselling, and long-term mental health care into emergency response programs, particularly in flood-prone regions.

## 2. Improve early warning systems and communication

The lack of early warning dissemination was significantly associated with heightened psychological distress. Therefore, investment in robust, inclusive, and community-sensitive early warning systems is critical. These should include both digital and non-digital methods to ensure accessibility for all socioeconomic groups.

## 3. Strengthen livelihood diversification and recovery support

Livelihood disruption was widespread, especially among those dependent on agro-fishery sectors. Programs promoting vocational training, microfinance, and small business support can enhance income diversification and reduce long-term vulnerability to flood impacts.

## 4. Enhance infrastructure and basic services

Efforts should be made to improve housing structures, access to safe drinking water, and sanitation facilities in flood-prone areas. Resilient infrastructure can significantly reduce both livelihood losses and health risks during future floods.

## 5. Target support to vulnerable populations

Special attention should be given to groups identified as highly vulnerable, such as individuals with chronic illnesses, low educational attainment, and residents of severely affected areas like Sonagazi. Tailored support packages, including cash transfers and community-based care, can address their specific needs.

## 6. Promote Community-Based mental health awareness

Community outreach programs that reduce stigma, raise awareness about mental health symptoms, and encourage help-seeking behaviour can play a pivotal role in early identification and intervention, especially in rural and marginalized communities.

## 7. Conduct longitudinal and comparative studies

Further research using longitudinal designs is needed to assess the long-term impacts of floods on both mental health and livelihoods. Comparative studies across different regions can help identify broader patterns and inform national disaster resilience strategies.

## Conclusion

The 2024 flash flood in Feni District, Bangladesh, had a profound impact on both the livelihoods and mental health of the affected population. The study revealed that almost all respondents experienced disruption to their livelihoods, with agricultural sectors particularly hard-hit. Psychological distress was widespread, with over 85% of participants reporting moderate to severe symptoms of distress, underscoring the significant mental health burden of the disaster. The regression analyses identified key sociodemographic factors, such as education, occupation, and income, as major influences on livelihood disruption. At the same time, age, marital status, and chronic illness were significantly associated with higher levels of psychological distress. The findings highlight the urgent need for integrated disaster response strategies that address both immediate economic recovery and long-term mental health support. Strengthening early warning systems, improving resource accessibility, and enhancing community-based mental health services are crucial steps toward building resilience in flood-prone areas. In addition, promoting livelihood diversification and enhancing infrastructure can reduce the vulnerability of communities to future disasters. This research contributes to the limited body of knowledge on the combined impacts of natural disasters on economic and psychological well-being, particularly in low-resource settings like Bangladesh. However, further longitudinal studies and comparative research are necessary to understand the long-term effects and inform more effective disaster management frameworks. The evidence presented provides a foundation for policymakers, practitioners, and researchers to develop targeted interventions that can mitigate the devastating effects of future floods on both livelihoods and mental health.

## Data Availability

The data generated from this study are used to write this research article and are embedded in the manuscript.
